# CD25+CCR4+ cells as a marker of HTLV-1-infected cells in peripheral blood

**DOI:** 10.1186/1742-4690-11-S1-P139

**Published:** 2014-01-07

**Authors:** Huseini Kagdi, Anat Melamed, Silva Hilburn, Maria A Demontis, Charles Bangham, Graham Taylor

**Affiliations:** 1 Infectious Diseases and Immunology, Faculty of Medicine, Imperial College, London, UK

## 

The diagnosis of Human T cell Lymphotropic Virus type 1 (HTLV-1) infection is based on serology and PCR. The burden of HTLV-1 viral infection is monitored by measuring the proviral load [PVL] in peripheral blood. This assay is not useful for isolation of HTLV-1-infected cells. HTLV-1 Tax upregulates CD25 expression on infected CD4+ T cells. However, CD4+CD25+ is also the phenotype of activated T cells. CCR4 is a chemokine receptor expressed on subsets of activated T cells. HTLV-1-infected cells secrete the CCR4 ligand CCL22, and preferentially infect CCR4+ Cells. We studied the expression of CD25 and CCR4 in lymphocytes and its relation to PVL. We performed 11-colour immunophenotyping and HTLV-1 PVL quantification on 53 samples obtained from 36 HTLV-1 infected patients, [10 asymptomatic carriers (AC); 11 patients with HTLV-1-associated myelopathy (HAM); 4 co-infected with HIV; 11 with chronic/smouldering adult T-cell leukaemia/lymphoma (ATL)] and 3 uninfected individuals. Increased frequency of CD25+CCR4+ T cells [median: 14.7%, range: 1.95-91.3%] was observed in all HTLV-1 infected patients; the frequency correlated with PVL [Spearman r=0.89, P <0.001, Linear R^2^ = 0.61]. CD4+ and CD8+ cells from 12 patients [6ACs and 6 HAMs] were separated for PVL estimation. CD25+ CCR4+ cell counts correlated closely with PVL in CD4+ cells [Spearman r=0.816, P <0.001, linear R^2^=0.886] but not in CD8+ cells. Frequency of CD25+CCR4+ T cells correlated with PVL change in treated ATL patients. We conclude that CD25+CCR4+ cells can be used as a specific marker for monitoring of HTLV-1 infection and isolation of HTLV-1 infected cells.

